# Clinical and Microbiological Profile of Urinary Tract Infections in Diabetic versus Non-Diabetic Individuals

**DOI:** 10.7759/cureus.5464

**Published:** 2019-08-22

**Authors:** Ravi Kumar, Rajesh Kumar, Prinka Perswani, Muhammad Taimur, Ali Shah, Faizan Shaukat

**Affiliations:** 1 Internal Medicine, Jinnah Sindh Medical University, Karachi, PAK; 2 Internal Medicine, Liaquat University of Medical and Health Sciences, Hyderabad, PAK; 3 Internal Medicine, Dow University of Health Sciences, Karachi, PAK; 4 Internal Medicine, Jinnah Postgraduate Medical Center, Karachi, PAK

**Keywords:** urinary tract infections, diabetic vs non-diabetic, escherichia coli (e. coli), pseudomonas aeruginosa, pakistan

## Abstract

Introduction

Diabetic patients have a higher tendency of developing all infections, especially infections of the genitourinary tract. In most cases, urinary tract infections (UTI) in diabetic patients are asymptomatic. The aim of this study to was to compare the incidence and clinical and microbiological features of UTI between diabetic and non-diabetic patients.

Methods

In this prospective, comparative study, the incidence and clinical and microbiological features of UTI were compared between diabetic and non-diabetic patients via consecutive non-probability sampling technique. For every diabetic patient, one non-diabetic control was included. All patients were screened for UTI through a midstream urinary sample. Their demographic characteristics, clinical profile, and urinary microscopy were compared. Data were entered and analyzed using SPSS version 22.0.

Results

In the diabetes group, 35/256 (13.67%) patients had culture-positive UTI as compared to 18/250 (7.2%) in the non-diabetic group. Diabetic group had twice the risk of UTI (*p *= 0.01; odds ratio [OR]: 2.04; confidence interval [CI]: 1.12, 3.71) and female gender in the diabetic group had a risk of almost five times (*p *= 0.01; OR: 4.93; CI: 1.12, 20.16) that of the non-diabetic group. In the diabetic group, 31.4% patients were asymptomatic as compared to 5.6% in the non-diabetic group (*p *= 0.03; OR: 7.79; CI: 0.92, 66.18). *E. coli* was the most commonly identified microorganism in both groups. *Pseudomonas aeruginosa* was identified in 14% of diabetic cases and none in the non-diabetic.

Conclusions

UTIs are more frequent among diabetics. Asymptomatic bacteriuria is a more common entity in diabetic patients and does not require any treatment.

## Introduction

Diabetes mellitus is associated with immune system dysfunction, which makes the affected individuals prone to repeated infections, especially infections of the genitourinary tract. Diabetic patients have more than twice the tendency of developing genitourinary tract infections [1]. The altered host responses in diabetic patients cause increased adherence of the microorganisms to the uroepithelial cells, granulocyte dysfunction, and altered intracellular calcium metabolism. This results in increased susceptibility of these individuals to develop urinary tract infections (UTI) [2-3]. Another important pathology underlying the increased incidence of UTIs and recurrent UTIs in the diabetic population is diabetic bladder dysfunction. It affects more than half of the people with longstanding and poorly controlled diabetes. It includes storage problems as well as voiding problems [4]. Furthermore, some pathogens flourish well in high glucose environment [5].

UTIs may be symptomatic or asymptomatic [6]. The common symptoms include burning micturition, urgency, dysuria, cramping in the lower abdomen, mental irritability, back or flank pain, chill, nausea, fever, vomiting, fatigue, and weakness [7]. *Escherichia coli* (*E. coli*) is the prominent causative pathogen of UTI in both diabetic and non-diabetic populations, followed by coagulase-negative *Staphylococci *(CONs), *Enterococcus* species (spp.), *Candida albicans*, and non-albicans *Candida* spp. [6,8]. It is very important to screen diabetic patients for UTI for timely diagnosis, complete treatment, and prevention of progression to renal complications and ultimately severe renal failure. However, there are controversies regarding the incidence, clinical pattern, and microbiology of UTIs in diabetic individuals as compared to those in non-diabetic ones [5]. Hence, this study was conducted to compare the incidence and clinical and microbiological features of UTIs between diabetic and non-diabetic patients. It would help assess the frequency of UTIs in the diabetic population and enable the diabetologists predict the clinical and microbiological patterns of UTI in their patients.

## Materials and methods

This prospective, observational, cross-sectional, comparative study was conducted from July 1, 2017 to February 28, 2018 in the outpatient department (OPD) of a general hospital in Pakistan. Ethical approval from the institute was acquired. Consecutive non-probability sampling technique was adapted. All patients of type II diabetes mellitus, of both genders and age 18 years and above, were recruited after informed consent. All patients were screened for UTI through a midstream 5-ml urinary sample. The presence of bacteria, positive leukocyte esterase, and white blood count (WBC) >5 per high power field (HPF) were taken as diagnostic for UTI. Pyuria was defined as WBC >10/HPF, and hematuria was defined as red blood cells >5/HPF. Urinary culture analysis, for identification of the pathogen, was performed only for patients who were found to be infective on urine microscopy.

Clinical and demographic characteristics included in the study were as follows: age, gender, and HbA1c for both groups. For the diabetic group, duration of diabetes, diabetes-related complications, and the presence of diabetic kidney disease were also noted. Patients who had taken antibiotics within the last two weeks for any reason were not included in this study. Patients with anatomical and neurologic urinary tract abnormalities, pregnant women, cases of complicated UTI (including pylonephritis), and patients with acute and/or chronic renal failure were also excluded.

During the study period, 260 diabetic patients were recruited. For every diabetic patient, a non-diabetic patient was included. The non-diabetic control group was selected from the attendants of the diabetic group to align their sociodemographic characteristics. By the end of the study, there were 256 records in the diabetes group and 250 records in the non-diabetes group. Their data was managed using SPSS for Windows version 22.0 (IBM Corp., Armonk, NY). The mean and standard deviation were calculated for continuous variables such as age, duration of diabetes, and HbA1c levels. Frequency and percentages were calculated for categorical variables. The incidence of UTI was compared for the diabetic and non-diabetic groups. Chi-square was applied for comparison. *P*-value ≤0.05 was taken as significant. Odds ratio (OR) and confidence interval (CI) were calculated.

## Results

Among 256 patients in the diabetic group, there were 112 (43.7%) males and 144 (56.3%) females. Their mean age was 56 ± 11 years. Non-diabetic patients were relatively younger with a mean age of 48 ± 12 years. There were more women (*n *= 156; 62.4%) than men (*n *= 94; 37.6%) in the non-diabetic group. Demographic and clinical characteristics of both study groups are compared in Table 1.

**Table 1:** Demographic and clinical characteristics of participants in the diabetic group (n = 256) and non-diabetic group (n = 250)

Patient Characteristics	Diabetic Group (n = 256)	Non-diabetic Group (n = 250)
Gender		
Male	112 (43.7%)	94 (37.6%)
Female	144 (56.3%)	156 (62.4%)
Age in years		
Mean	56 ± 11	48 ± 12
Less than 40 years	38 (14.8%)	72 (28.8%)
40-60 years	115 (44.9%)	87 (34.8%)
Above 60 years	103 (40.2%)	91 (36.4%)
Duration of diabetes in years		
Mean	7.6 ± 3.8	Not applicable
Less than 5 years	78 (30.5%)	
5-10 years	97 (37.8%)	
More than 10 years	81 (31.6%)	
Diabetic complications (any)		
Yes	118 (46.1%)	Not applicable
No	138 (53.9%)	
Diabetes-related kidney disease		
Yes	34 (13.3%)	Not applicable
No	222 (86.7%)	
Glycosylated haemoglobin A1c (%)		
Mean	7.8 ± 2.6	4.9 ± 1.2
Less than 7%	48 (18.7%)	250 (100%)
7%-8.5%	134 (52.3%)	Not applicable
More than 8.5%	74 (28.9%)	

In diabetes group, 35 (13.67%) patients were identified with culture positive UTI as compared to 18 (7.2%) participants in non-diabetic group. In both groups, UTI was more common in female gender. Diabetic group had an overall twice risk of UTI (*p* = 0.01; OR: 2.04; CI: 1.12, 3.71) and female gender in diabetic group had a risk of almost five times (*p* = 0.01; OR: 4.93; CI: 1.12, 20.16) that of the non-diabetic group of developing urinary tract infection (Table 2).

**Table 2:** Incidence of urinary tract infection in the diabetic group (n = 256) and non-diabetic group (n = 250)

	Diabetic Group (n = 256)	Non-diabetic Group (n = 250)	P-value	Odds Ratio	Confidence Interval
Total	35 (13.7%)	18 (7.2%)	0.01	2.04	1.12, 3.71
Male	4/35 (11.4%)	7/18 (38.9%)	0.01	0.2	0.05, 0.83
Female	31/35 (88.6%)	11/18 (61.1%)		4.93	1.12, 20.16

Almost 30% patients in the diabetic group with culture-proven UTI were asymptomatic as compared to only 5% in the non-diabetic group (*p* = 0.03; OR: 7.79; CI: 0.92, 66.18). There was no other significant difference between the presentations of UTI in the two groups, as shown in Table 3.

**Table 3:** Clinical and incidence of urinary tract infection in the diabetic group (n = 256) and non-diabetic group (n = 250)

Signs / Symptoms	Diabetic Group (n = 35)	Non-diabetic Group (n = 18)	P-value	Odds Ratio	Confidence Interval
No signs / symptoms	11 (31.4%)	1 (5.6%)	0.03	7.79	0.92, 66.18
Fever	21 (60.0%)	11 (61.1%)	0.93	0.95	0.3, 3.06
Dysuria	17 (48.5%)	11 (61.1%)	0.38	0.6	0.19, 1.91
Increased frequency (≥5/day)	13 (37.1%)	7 (38.9%)	0.90	0.93	0.29, 2.99
Dribbling	9 (25.7%)	5 (27.8%)	0.87	0.9	0.25, 3.24
Abdominal / flank pain	9 (25.7%)	5 (27.8%)	0.87	0.9	0.25, 3.24
Pyuria	7 (20.0%)	2 (11.1%)	0.41	2.0	0.37, 10.81
Vomiting	4 (11.4%)	2 (11.1%)	0.97	1.03	0.17, 6.25
Urinary retention	4 (11.4%)	2 (11.1%)	0.97	1.03	0.17, 6.25
Hematuria	2 (5.7%)	1 (5.6%)	0.98	1.03	0.09, 12.19

*E. coli* was the most commonly identified microorganism in both diabetic and non-diabetic groups. *P. aeruginosa* was identified in 14% of diabetic cases. Other organisms included *Klebsiella* species and *Enterobacter* species (Table 4).

**Table 4:** Microorganisms identified in the diabetic group (n = 35) and non-diabetic group (n = 18) on urine culture

Organisms	Diabetic Group (n = 35)	Non-diabetic Group (n = 18)	P-value	Odds Ratio	Confidence Interval
Escherichia coli	21 (60.0%)	13 (72.2%)	0.37	0.58	0.17, 1.98
Klebsiella species	6 (17.1%)	2 (11.1%)	0.56	1.66	0.3, 9.18
Enterobacter species	3 (8.6%)	1 (5.6%)	0.69	1.59	0.15, 16.52
Coagulation-positive Staphylococcus	2 (5.7%)	2 (11.1%)	0.48	0.48	0.06, 3.76
Candida albicans	3 (8.6%)	----	Not applicable		
Pseudomonas aeruginosa	5 (14.3%)	----			
Coagulation-negative Staphylococcus	12 (34.3%)				

## Discussion

This study compared the incidence of UTIs in demographically comparable groups of diabetic and non-diabetic individuals. There was an overall significantly higher incidence of UTIs in the diabetic group; these individuals had twice the risk as compared to non-diabetics. Females also showed a significantly higher incidence of UTIs in the diabetic group. Females had an overall five-time higher risk of developing UTI in the diabetic group. There were no stark differences in the clinical and microbiological profiles of these patients; however, the diabetic group showed significantly more patients with asymptomatic UTI.

This study has provided substantial evidence to the comparatively higher risk of UTI in diabetic patients. However, it has its limitations too. This study was conducted in the OPD and only included clinically stable outpatient cases; hence, many cases with complicated UTI must have been missed. This study did not include the antibiotic sensitivity profile for both groups.

Previous studies reported the incidence of UTI in Pakistani diabetic patients to be 50% to 53% [9-11]. These figures are higher than those obtained in our study (13.7%). In a Romanian study, the prevalence of UTIs in patients with DM was 12% [12]. An Indian study deduced the prevalence of asymptomatic bacteriuria to be significantly higher (28%) among diabetic patients as compared to non-diabetics (7.5%; *p* = 0.001) [13]. Higher incidence of UTI among females in the diabetic group as wells as in the general population has been reinforced in various studies [9-13]. In a study that compared the pattern of UTI in diabetic and non-diabetic females, it was seen that uncontrolled diabetes was associated with increasing severity of UTI. *E. coli* was the most commonly isolated pathogen in both groups. *Candida* was only seen in diabetic females group [14]. *E. coli *remained the most common pathogen in both groups of this study. Only 5% of cases of *Candida* were reported in the diabetes group. *Pseudomonas* was also only reported in the diabetes group in this study. Diabetics individuals are in a immunosuppressed state, hence at a greater risk of contracting *Pseudomonas* infection. Compared to the incidence of *Pseudomonas* in this study (14%), other studies from Pakistan have reported varied incidence. Ijaz et al. reported that among diabetics, 72% urinary samples were positive for *Pseudomonas;* Bashir et al. reported that 1% cases of *Pseudomonas* were isolated, and Zahra et al. 6% cases of *Pseudomonas* were isolated from non-diabetic urinary samples and none from the diabetic population [9-11]. In non-diabetic Pakistanis, 5% urinary samples were found to be positive for *Pseudomonas* [15]. In an Indian diabetic sample of 651 culture-positive UTI, the frequency of *Pseudomonas* was 2.7%; similar to our study, *E. coli* was also the most commonly isolated pathogen (69%) [16].

Asymptomatic pyuria was significantly more common in the diabetic group as compared to non-diabetic in this study (30% vs. 5.6%; *p *= 0.03). In an Indian study, asymptomatic bacteriuria was found in 40% of urinary samples in a diabetic population. Hematuria was reported in 4% of their samples, as compared to 5.7% in our diabetic samples. The most common isolate in their study was also E. coli [17]. In a Sudanese diabetic sample, the frequency of UTI was 19.5%. Asymptomatic bacteriuria was present in 21% of these patients [18]. Clinical presentation of UTI in both groups was comparable in our study. Similarly, no significant difference was seen in the clinical presentation in Aswani et al. [5]. Even the frequency of asymptomatic bacteriuria was similar (30%) in both diabetic and non-diabetic groups in their analysis [5]. According to the Infectious Disease Society of America (IDSA) guidelines, diabetic patients should not be screened or treated for asymptomatic bacteriuria [19]. When clinical signs are present, UTIs are to be treated as per the culture and sensitivity report.

## Conclusions

The frequency of UTIs is higher in the diabetic population as compared to their non-diabetic counterparts. UTIs are more common among females in both groups. Clinical presentation in the two groups is also similar. Asymptomatic bacteriuria is a more common entity in diabetic patients and does not require any treatment.
